# Evaluation of an Integrative Care Program in Pediatric Oncology

**DOI:** 10.1177/1534735420928393

**Published:** 2020-07-10

**Authors:** Wiebke Stritter, Britta Rutert, Angelika Eggert, Alfred Längler, Christine Holmberg, Georg Seifert

**Affiliations:** 1 Charité—Universitätsmedizin Berlin, Berlin, Germany; 2 Gemeinschaftskrankenhaus Herdecke, Witten/Herdecke University, Herdecke, Germany; 3 Institut für Sozialmedizin—Brandenburg Medical School Theodor Fontane, Brandenburg, Germany

**Keywords:** pediatric oncology, integrative care program, rhythmic embrocation, oil-compresses, qualitative study, evaluation

## Abstract

**Purpose:** This article discusses the results of an evaluation of the one-year implementation period of an integrative care program at a pediatric oncology ward, which consists of integrative care treatments offered three times a week to the patients. The guiding questions are how the model was implemented, which factors have to be considered for successful implementation, and which factors showed to be obstacles during implementation. **Methods:** A mixed-methods approach was applied for data saturation. Qualitative data consist of participant observations and informal conversations during the implementation phase. All observational records were filed in the data program MAXQDA. For the quantitative data, all integrative care treatments applied on the intensive care unit were documented and subsequently filed in an Excel sheet. Both sets of data were analyzed for the evaluation. **Results:** Four main thematic clusters influenced the implementation: (1) the organization and structure of the intensive care unit; (2) mood and atmosphere; (3) feedback on treatment; and (4) time and experience. All factors are interlinked and cannot be looked at independently. Results of the quantitative data show that the most frequent used treatments were those with calming and relaxing effects, followed by treatments for stomachache, nausea, and obstipation. **Conclusions:** The implementation of an integrative model of care is a process that demands thorough understanding of the complex setting of the ward, ongoing adaptation to the structures and organization of the ward, and the integration of factors like feedback, time, atmosphere, and the mood of parents, patients, and nurses.

## Introduction

In the past years, the use of traditional and complementary medicine (T&CM) and care (formerly referred to as alternative and complementary medicine [CAM]) has generally increased.^1,2^ To manage the physical, emotional, social, and spiritual impact of a cancer diagnosis, people often seek T&CM modalities.^2-4^ Adaptations to patients’ needs have resulted in an increase of T&CM offers in clinical settings.^5-7^ However, because of their differing cultures, philosophies, historical development, and settings, integrating T&CM modalities into hospital settings may be challenging.^8-10^ Different models to integrate T&CM modalities into conventional medicine and care have been developed since the 1990s,^11^ and some hospitals, clinics, and pediatric wards in Europe and the United States have begun to offer them.^12,13^

Whereas integrative medicine and care are slowly finding their way into the German hospital landscape,^3^ university hospitals still have an ambivalent attitude toward T&CM.^14,15^ There is growing research on supportive T&CM interventions for specific symptoms in pediatric oncology such as fatigue, psychological stress, and pain,^16-18^ and also few descriptions of implementation projects in pediatric settings.^19,20^ To date, however, limited research on the implementation, applicability, and evaluation of integrative care in the highly specialized field of children’s oncology is available in Germany.

One reason may lie in the complexity of the intervention required to incorporate integrative care elements into conventional care on the one hand, and the complexity of the setting on the other.^21^ According to the guidance of the Medical Research Council, a complex intervention is “built up from a number of components, which may act both independently and interdependently.”^22,23^ A complex intervention requires a degree of flexibility or tailoring.^22^ Accordingly, the successful implementation of integrative care into daily care requires thorough understanding of the particular components at work and the roles they play in the implementation process.

This article describes the results of the evaluation of the implementation of a context-specific and patient-focused integrative care program in the pediatric oncology ward of a university hospital in Germany. It looks at the components defining both the success and limitations of the implementation of the program, which is part of a larger third-party-funded project, which aims to incorporate integrative care elements into daily care. A description of the planning and development of this particular integrative care program has been published earlier.^24,25^ This article inquiries into how the integrative care program was implemented in daily care and which components played a role in the success of implementation.

## Methods

### Study Design

Over the course of 1 year, the integrative care program was implemented and evaluated on a pediatric oncology ward at Charité Universitätsmedizin, Berlin, Germany. This ward is structured as an intensive care unit (ICU). A mixed-methods approach was undertaken for the evaluation. We received ethical approval from the Ethical Committee of the Charité—Universitätsmedizin Berlin (EA2/132/16). Written informed consent was obtained from all study participants included in the study.

The program was developed on the basis of anthroposophic care interventions and was tailored to the specific needs of the pediatric oncology patients as well as the structural and time resources of an ICU. A detailed description of the developmental process is published elsewhere.^25^ It consisted of three 8-hour integrative shifts per week (Monday, Wednesday, and Friday). These were offered on the ward in a rotating basis by 1 of a team of 5 nurses, all of whom were trained in integrative care. The integrative nurse offered warm oil compresses and rhythmic embrocation with essential oils to all patients on the ward (with permission of their caretakers). Concurrently, the five integrative nurses received regular training in integrative care outside the ward to enable the professional sustainability of the program on the ward. Moreover, to enable the sustainability of the program on the ward, other nurses and patient guardians were trained in integrative care treatments by the integrative nurses during the integrative shift.

To answer our research question regarding which components were important for the successful implementation of an integrative care program on a pediatric oncology ward, we used both qualitative and quantitative data collection methods.

### Data Collection

For the evaluation of the program, the implementation process was documented using different methods, which included participant observations that were recorded in field notes, and documentation of the treatments offered on the ICU. Documentation of the treatments covered the following: (1) which patient was treated, (2) diagnosis of the patient, (3) symptom claimed by the patient, (4) treatment offered, and (5) the oil used. All treatments were first documented by the applying nurse in an extra folder and then transferred into an Excel sheet for descriptive analysis. The training of parents and additional nurses during the integrative shift was also documented in an extra documentation sheet, and also transferred into an Excel sheet. The nurses who gave the treatments collected these data. The participant observation was conducted by experienced qualitative researchers: first an anthropologist (BR), who conducted the observations, and then an anthropologist and psychologist (UA), who took over for BR. Participant observation is a scientific method of data collection deriving from ethnographic research. In a planned manner, observations about everyday practices like care practices and human interactions are documented in detailed field notes.^26^ The participant observation focused on the integrative nurse practices during the integrative shifts, with particular concentration on the integrative care interventions carried out during those shifts including interactions with and reaction of the patients and caregivers. All citations in the article are taken from observational records. To maintain the anonymity of the data secured, we make no reference to the corpus of data in the results.

### Data Analysis

Field notes were pseudonymized and transferred into the data management program MAXQDA for analysis. The field notes were coded and analyzed on the basis of thematic analysis.^27,28^ The codes were grouped, paraphrased, and reduced to thematic clusters. Two different researchers (BR and CE) with experience and training in qualitative analytic methods completed the coding. Authors BR, the main researcher, and CE, a researcher who was not involved in the participant observations, independently coded the observational records. CH and WS reviewed the initial clusters for thematic accuracy. Descriptive statistics using Excel were conducted on the collected data on treatments.

## Results

### Quantitative Results

Over the course of the evaluation period, the integrative nursing team documented 640 times that they offered patients integrative care (with permission of their caretakers). Of these 640 offers for integrative applications, the patients agreed 340 times in total to receive the treatment (total offers). Many patients (n = 69) were offered and received the treatments more than once, especially long-term patients. The total number of individual patients who were asked was 134. Of these 134 patients, 98 agreed to the treatment, resulting in a response rate of 72.06% of the total patients. A detailed description of the treatments can be found in Table 1.

**Table 1.** Integrative Care Treatments.

	Treatment offers made	Treatment offers accepted
Total offers	640	340
Total patients (1-25 uses)	136	98
Male	85	58
Female	51	40
No acceptance	38	
1-2 acceptances		53
3-9 acceptances		39
10-25 acceptances		6

**Figure 1.**
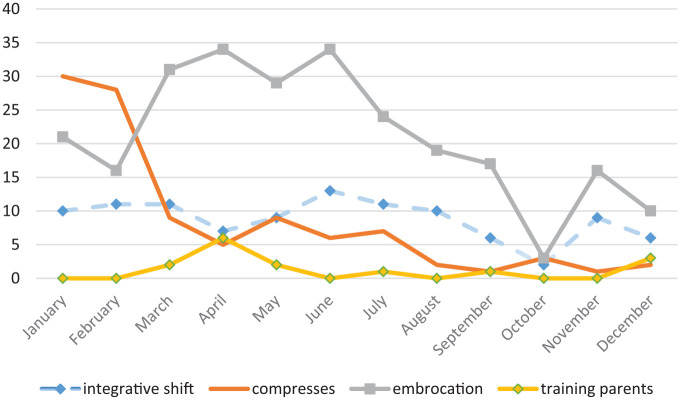
Development of integrative care treatments over time.

In total, 105 integrative shifts were carried out. The rate per month varied from two to 13 shifts. While the number of shifts per month was high at the beginning of the implementation phase, this changed toward the end, when the number reduced. Due to a deficit of nursing staff overall in the nursing team, a resignation of the integrative nursing team resulted in the second half of the year when integrative shifts had to be cancelled to ensure understaffed regular shifts. The training of parents, as presented in Figure 1, was relatively low: 21 trainings were documented in total. However, it was observed during the participant observation in the qualitative part of the evaluation that trainings also occurred informally at patients’ beds and were not always documented in the formal documentation sheet.

The number of treatments, types of oils, patient-stated symptoms, and the aims of the treatments applied are depicted in Table 2. The most frequently used oils were gold-rose-lavender oil and lavender oil. This was based on the most often stated symptoms and resulted in treatments with a calming, relaxing effect. Other oils like milfoil or fennel-caraway, used against stomachaches, nausea, and obstipation, commonly known side effects of chemotherapy,^29^ were also in high demand.

**Table 2.** Symptoms, Used Oils, and Body Region of the Integrative Care Treatments.

Symptoms/aim of treatment	Number	Oil/active substance	Number	Body region	Number
Relaxation, calming, well-being	160	Gold-rose lavender	69	Stomach	152
Stomach ache, obstipation, nausea, digestion	74	Lavender	62	Back	145
Pain (head, back, other regions)	41	Yarrow	44	Feet/calves	58
To aid sleep	19	Fennel-Caraway	38	Neck/shoulders	18
Revival, refreshment, strengthening, warming	16	Balm	37	Chest	9
Training of parents	22	Aconite	36	Knees	2
Tension	6	Arnica	22	Throat	1
Care, trust, distraction	4	Citrus	20	Head	1
Lung issues	4	Calendula	16	Not specified	10
Dry skin	2	Hollyhock	9		
Chemotherapy side effects	2	Cetaphil	8		
Not specified	92	Linola	3		
		Intervention without oil	2		
		Solum	1		
		Rosemary	1		
		Not specified	19		

### Qualitative Research Results

Analysis of the observational records yielded 4 thematic clusters that had both a positive as well as a challenging impact on the success of the implementation: (1) the organization and structure of the ICU; (2) mood and atmosphere; (3) feedback on treatment; and (4) time and experience. All thematic clusters are connected and interlinked.

#### Organization and Structure of the ICU

The implementation of the project was determined by the structure and organization of the ICU. Beyond the general organization of the ward, one key impact was the lack of nursing staff members and understaffed shifts. As a result, the integrative nurses were repeatedly assigned within the regular shift system, either on the ICU or on other wards within the clinic. The head of the ward often communicated this on short notice. This led to resentment and frustration among the integrative nursing team:

What really irritates me is when the position simply isn’t taken seriously, and one has to fight for it. Last week, when they sent me to [another ward]. I found that pretty irritating

If [nurse] T has her study nurse duties they [head of the ward] are not going to put her in a regular shift. Why is this different with the integrative shift?

In addition to the recurring shift changes, a number of internal structures, like the serving of lunch, ward rounds, and other therapeutic treatments, challenged the implementation of the integrative shift. This resulted in the integrative nurses often having spare time during the 8-hour shift, which led to a feeling of unease. Particularly when the regular shifts were busy, the integrative nurses felt remorse:

When everyone’s running around like crazy, then I feel lazy if I am only doing integrative care. That’s simply not right.

The integrative nurses’ unease was fostered by derogatory terms used for them in the beginning by some of the general nursing team, such as “*Läppchen-Schwester*” (rag nurse) and “*Zwischenwischen-Dienst*” (smear between shift). To avoid these negative attributions and the uneasy feelings, the integrative nurses often organized the integrative shift along 2 tasks: (1) applying integrative care and (2) helping out on the ward during regular shifts. Especially when the regular shifts were understaffed, the regular nurses appreciated the help of the integrative nurse. One integrative nurse explained:

I don’t feel comfortable with this extra role as integrative nurse. That’s why I did a parental discharge talk during the shift and took a patient to surgery.

#### Mood and Atmosphere

Most of the time, however, the nurses enjoyed the integrative shifts. In particular, the atmosphere created in the patients’ rooms during a treatment had a positive impact on the perceptions of the treatments:

She [the mother] explained to her daughter what [nurse] D wanted to do, and asked her if she wanted that. She said yes, but with a bit of reservation. D began with the stomach massage. Slow calm circles around the bellybutton. It became calmer in the room. As is so often the case with embrocation, this “sacred rest” enters, a calm, in which time and space cease to exist, and you only feel the interaction between the caregiver and patient, or the energy from the embrocation. The young patient seemed to enjoy it, despite her initial reservations. Her gaze slowly softened.

The integrative nurses said they particularly enjoyed applying the embrocation, as these included time, touch, and talking with patients and parents, moments often missing in daily care. With the beginning of a rhythmic embrocation, the patients fell into silence; they relaxed and felt the rhythmic touch applied by the nurse. Patients would fall asleep and the room would become silent. Parents also became hushed; they would sit next to the patient’s bed and observe the scene supportively, or they left the room to leave the nurse and the patient alone. On other occasions, patients did not want to be treated at all; either they were not in the mood to be touched, they did not feel well, they were simply not interested, or they preferred to be massaged by their parents. For this reason, the training of parents was a central aspect of the integrative shift. The trainings took place at the patient’s bed, before, during, or after an actual treatment. Parents would observe how to apply the treatment and would then be shown the specific technique by the integrative nurse. The embrocation in particular helped parents to get into close contact with their children and to support them in the convalescence process during their exhaustive chemotherapeutic treatments. One mother commented:

This is wonderful, and my son loves the foot massage. Every evening I massage his feet so that he can sleep.

#### Feedback

The feedback of patients played a central role in the implementation phase. The integrative nurses offered a treatment to every patient, who could voluntarily choose whether they wished to get a rhythmic embrocation or a warm oil compress. Some patients were hesitant at first, but changed their minds once they had experienced the first treatment. Often this was dependent on the length of their stay in the ward. Some patients only stayed for 1 to 3 days, while others stayed for many weeks. Long-term patients in particular seemed to profit from and enjoyed the relaxing and soothing effects of the treatments as some of them repeatedly asked for treatment or agreed to it when asked. Parents also were central to the decision of whether patients accepted or refused the treatment. They would support their children in trying the treatment. As one parent put it:

Try it once, it’ll be better than whatever you’ve already experienced here.

Most often, the patients themselves commented after the embrocation that it had been very nice or that they wished the nurse to continue and not stop. With the positive feedback of patients, and the support of parents, awareness of the treatments increased in the ward. Over time, the use of derogatory terms by other nurses, such as “*Läppchen-schwestern*,” diminished and acceptance of the integrative shift increased. One integrative nurse said:

Now slowly the other [nurses] respect that I’m doing an integrative shift. They no longer ask me so often if I can help out. The integrative shift is being increasingly accepted as an independent shift.

#### Time and Experience

Time was always scarce, especially during understaffed shifts. Even though the other nurses on the ward also wished to learn and apply integrative care themselves, they did not have the time to do so.

I would love to learn the application [of integrative methods], but I simply don’t have the time for it during my [normal] shift.

This is despite the fact that the actual time needed for the preparation and application of a treatment was not necessarily that great. The preparation of an oil compress, for instance, took 2 minutes; an embrocation can last between 2 and 20 minutes. Applying one treatment therefore did not take much time, and could be smoothly integrated into daily care. And yet the regular nurses hardly ever applied integrative care during their normal shifts. The integrative nurses nevertheless regarded the extra time spent with the patients as very positive and with growing experiences, also gained with the ongoing training in integrative care outside the ward, they appreciated the treatments even more. One nurse commented:

It’s also very nice to have such a relaxed shift from time to time, to have time for the patients, to talk a bit with the parents.

On the other hand, the integrative nurses also perceived the length of the shift as a challenge, even though it offered plenty of time for the patients. Due to the structure of the working schedule of the ward, it was not possible to shorten the shifts.

## Discussion

**Figure 2.**
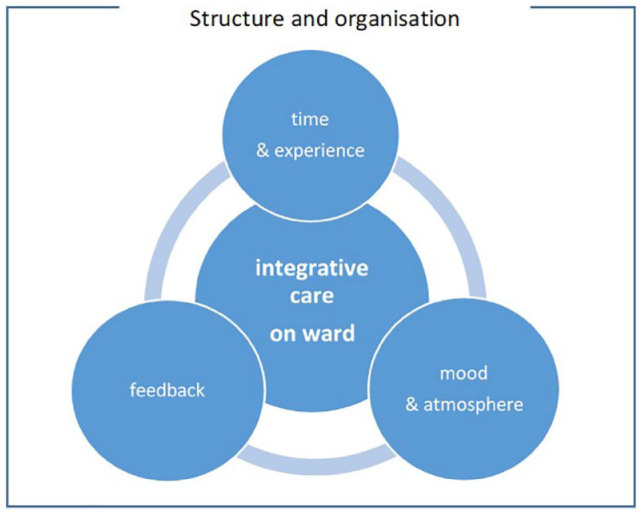
Interrelation of the thematic clusters.

This article presents the results of an evaluation of the implementation of an integrative care program at the pediatric oncology ward of a university hospital in Germany. The evaluation shows that 4 clusters played a vital role in program implementation: (1) the organization and structure of the ICU; (2) mood and atmosphere; (3) feedback on treatment; and (4) time and experience. Due to its multiple interacting and interdependent components,^8^ as well as the need for flexibility and adaptation to the context, the observed program and its implementation can be framed as a “complex intervention.”^30^ In addition, the ward itself can be called a complex system.^31^ With interventions in complex systems, nothing can be assumed constant as everything is linked to everything else.^21^ The interlinked clusters (Figure 2) that were detected in the program evaluation will be discussed hereafter.

The length of the implementation phase increased experience with and awareness of integrative care among nurses, parents, and patients. With growing experience, and with additional training outside the ward, the self-esteem and confidence of the integrative nurses grew. This was apparent in the use of rhythmic embrocation, which increased in terms of the number administered over the year, while the application of oil compresses decreased. Rhythmic embrocation builds on a specific technique^32^ and involves close and intimate contact with the patient. While the regular nurses, when they applied treatments, preferred to offer oil compresses to patients, the integrative nurses, with growing experience over time, prioritized the application of rhythmic embrocation. Most patients wished for a rhythmic embrocation for relaxation, followed by treatments for stomachache, nausea, and obstipation. The essential oils were chosen accordingly. Staying on a ward on a long-term basis leads to side effects such as back pain from lying in bed for a long time, and obstipation and stomachache from long-term chemotherapy and lack of physical movement.^29^ Long-term patients would therefore receive more treatments, while short-term patients received fewer treatments. Irrespective of the length of stay, the patients and parents recognized the calming effect of a rhythmic embrocation and the warmth of an oil compress. The mostly positive feedback on the treatments, made by patients and parents, resonated positively within the nursing team as a whole.

Voss and Kreitzer reported similar experiences from implementing Integrative Nursing at a pediatric blood and bone marrow transplant program in the United States.20 They also highlighted the aspects of the increase of integrative care over time and attributed this among other things to the increased demand from patients and parents.

The training of parents was an important part of the integrative shift. Most parents were supportive of the program and thus wished to be trained in the treatments. Due to the often understaffed shifts and the lack of time, the integrative shift could often not be applied. Parents, when trained, could continue with the treatments, either on the ward or at home. This was important in cases where patients did not want to be touched by “strangers” and preferred an embrocation by their parents. Children are different from adult patients. Some were simply not in the mood to receive a treatment. Initial mistrust, however, often turned into a positive reaction after the first treatment.

Furthermore, the nurses themselves had different moods concerning the treatments. A shift that can be organized without many instructions leaves space for personal preferences, moods, and decisions. Although the integrative nurses mostly enjoyed having extra time for the patients, they sometimes did not feel like doing the integrative shift because of the extra role they had on the ward. This mixture of structural, organizational, and personal constraints explains why (only) 640 treatments were documented in 1 year; why of these offers only 340 treatments were accepted by patients; and why only a total of 98 patients received treatments. Since the moods and movements of patients and nurses on the ward were unpredictable, the training of parents was identified as a key component of the integrative care program, also to sustain its sustainability.

### Obstacles to Implementing the Observed Integrative Care Program

During the evaluated implementation phase, the pediatric oncology ward was highly affected by a shortage of nursing staff. This was not only an issue on the pediatric oncology ward, but is a serious issue in the German health care system as a whole.^33^ The shortage of staff members and the resulting understaffed shifts created an atmosphere of stress and frustration on the ward. Under these conditions, resistance to all kinds of additional work impeded the implementation of this new program. This is one reason why the objective to train all nurses on the ward in integrative care treatments failed to some extent. The training of parents is key in circumventing the problem of nurses’ limited time.

In addition, the study described here was third-party funded. Without such initial funding, it may be difficult to implement a similar project at another university hospital in Germany. The same challenge has been reported by Eckert et al.^19^ While anthroposophic external applications are reported to be safe and used by patients with high satisfaction,^34^ they are often still less financially and institutionally supported by the German Health Insurance System.^15^ Substantive barriers—including economic, organizational, and scientific differences, as well as an apparent widespread lack of understanding—continue to hinder attempts toward integrative care.^35^ Ongoing costs for the program sum up to the costs of the materials, mainly the essential oils. A final economic analysis of the program, however, still needs to be executed.

### Recommendation for the Implementation of an Integrative Care Program

1. *Consider enough time for the implementation phase*: With time, the new treatments will be accepted by the nursing team and awareness of the treatments among parents and patients will increase.
2. *Acknowledge the initial resistance of nurses to additional work*: The positive impact of the treatments, the feedback of patients and parents, and the length of the project may change this resistance.
3. *Children are different from adult patients*: They have their own wishes, temperaments, and moods. These wishes and moods should be taken into account.
4. *Train the parents of patients*: They are the main care providers during the chemotherapeutic treatment and at home, and they often have more time for care than nurses.
5. *Provide simple treatments*: The treatments offered during the observed program were simplified versions of the original treatments and they were adapted to the specific conditions of the ward.
6. Establish a team of nurses among the nursing team to take responsibility for the program.
7. *Train as many nurses as possible on the ward*: The more nurses who are trained and who have experience with the treatments, the more integrative care will be accepted among the nursing team.
8. *Provide enough initial funding for the program implementation*: The training of nurses and the costs for materials like oils need to be guaranteed.
9. *Cooperate with the respective leaders such as the head of nursing on the ward*: The more cooperation there is on an institutional and personal level, the more acceptance integrative care will receive.
10. *Keep the program simple*: University hospitals are complex institutions. Keeping the program as simple as possible will enhance the chances of sustainable implementation.

## Conclusion

This evaluation shows that integrative medicine and care can be established in a pediatric oncology ward of a university hospital in Germany, if there is a structured program, sufficient time, acknowledgement of the specific structure and conditions of the ward, a team of responsible nurses, enough initial funding, and training modalities for parents and nurses. Aspects such as the moods of patients and nurses, the atmosphere created by the treatments, and the feedback thereof are simultaneously supportive of and a challenge for the successful implementation of such a program.
